# The Egyptian Dementia Network (EDN): Baseline characteristics from the first dementia registry in an African Arab country

**DOI:** 10.1002/alz.70770

**Published:** 2025-11-11

**Authors:** Shimaa A. Heikal, Gharib Fawi, Eman M. Khedr, Mai Othman, Sara A. Moustafa, Nesma G. Elsheikh, Heba M. Tawfik, Sara Elfarrash, Samer Salama, Eman M. Ali, Hany I. Hassanin, Mohamed Salama

**Affiliations:** ^1^ Institute of Global Health and Human Ecology The American University in Cairo Cairo Egypt; ^2^ Neurology Department Faculty of Medicine Sohag University Sohag Egypt; ^3^ Neuropsychiatry Department Assiut University Assiut Egypt; ^4^ Geriatric Medicine Department Faculty of Medicine Ain Shams University Cairo Egypt; ^5^ Medical Physiology Department Faculty of Medicine Mansoura University, and Medical Experimental Research Centre (MERC) Mansoura Egypt; ^6^ Neurology Department Faculty of Medicine Mansoura University Mansoura Egypt; ^7^ Community Nursing Department Faculty of Nursing Beni‐Suef University Beni‐Suef Egypt; ^8^ Atlantic Senior Fellow of Equity in Brain Health Global Brain Health Institute (GBHI), Trinity College Dublin Dublin Ireland; ^9^ Clinical Toxicology Department Faculty of Medicine Mansoura University Mansoura Egypt

**Keywords:** Alzheimer's disease, dementia, diagnosis, Egypt, environmental exposure, low‐ and middle‐income country, LMIC, multimorbidity, registry, risk factors

## Abstract

**INTRODUCTION:**

Dementia is a growing public health challenge in low‐ and middle‐income countries (LMICs) like Egypt, where data are scarce. The Egyptian Dementia Network (EDN) registry addresses this gap by capturing epidemiological, clinical, and environmental data across Egypt.

**METHODS:**

In this multicenter study, 662 participants from six governorates were enrolled using standardized tools.

**RESULTS:**

The cohort had advanced age (mean 68.3 years), low education (65.9% illiterate), and high comorbidities including hypertension (55%) and diabetes (23%). Alzheimer's disease (62%) and vascular dementia (23%) predominated. Only 24.4% received pharmacological treatment and 2.1% psychosocial support, highlighting care gaps. Household insecticide exposure (20.4%) was notable.

**DISCUSSION:**

EDN demonstrates the feasibility of implementing a national dementia registry in LMICs, generating baseline insights into demographic, clinical, and environmental risks. In addition, registry‐linked biosamples have enabled pilot multi‑omics and exposome analyses, underscoring its potential as a scalable scientific platform for future dementia research.

**Highlights:**

- Established Egypt's first national, multicenter dementia registry.
- Aimed to characterize dementia profiles and care gaps across diverse regions.
- Identified late‐stage diagnosis and limited access to dementia interventions.
- Uncovered unique environmental risk factors relevant to the Egyptian context.
- Provides a foundation for policy, research, and improved dementia care in Egypt.

## Graphical abstract

Overview of the Egyptian Dementia Network (EDN) registry highlighting multiple centers’ inclusion, cohort demographics, dementia diagnosis, and interventions.

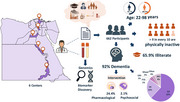

## 1 INTRODUCTION

Dementia, particularly Alzheimer's disease (AD), represents a critical and growing public health concern worldwide, with 70% of cases projected to occur in low‐ and middle‐income countries (LMICs) by 2050.^1^ Despite this, epidemiological research remains disproportionately focused on high‐income countries (HICs), where homogeneous cohorts dominate studies on risk factors, biomarkers, and care models.^2^ This creates a significant global disparity: HIC‐centric frameworks fail to address LMIC‐specific challenges, such as low literacy rates, fragmented healthcare systems, and culturally distinct caregiving practices.^3^

The increasing burden in LMICs is further compounded by systemic barriers to detection, diagnosis, and support.^4^ Over 90% of individuals with dementia in LMICs are undiagnosed, resulting in a lost opportunity for timely intervention and management.^5^ Conventional cognitive screening instruments, such as the Mini‐Mental State Examination (MMSE), suffer from poor validity in low‐literacy and culturally diverse settings, exacerbating underdiagnosis and misclassification.^6^ In addition, emerging evidence highlights environmental and exposomic risk factors, heavy metals, pesticides, and air pollution as contributors to dementia.^7^, ^8^ These remain understudied in LMICs, particularly in Africa and the Arab world.^4^, ^9^ This knowledge gap extends to clinical care as well, with psychosocial interventions and pharmacological treatments reaching only a small minority of patients, thereby reinforcing health inequities and constraining informed policy responses.^10^, ^11^, ^12^

Egypt, with high illiteracy rates, reliance on informal caregiving, and environmental vulnerabilities, provides a unique setting to investigate these relationships. Although the country's elderly population is rapidly increasing, national data on dementia prevalence and care remain scarce and fragmented.^4^ Existing studies report considerable fluctuations in dementia prevalence, ranging from 1% to nearly 6% across different regions and time periods^13^, ^14^, ^15^, ^16^, ^17^, ^18^, ^19^, ^20^ (Table 1). This variability is likely attributable to differences in study design, diagnostic criteria, and the sociodemographic characteristics of the surveyed populations. Notably, most studies have been limited to specific governorates or less densely populated areas, rather than being nationally representative.^21^ There is also limited information on comorbid conditions, environmental exposures, and the social determinants that shape disease risk and experience.^22^ As a result, the true burden of dementia in Egypt remains unclear, underscoring the urgent need for large‐scale, comprehensive epidemiological studies that reflect the country's diverse population.

**TABLE 1:** Studies reported dementia prevalence in Egypt.

Study	Period	Area in Egypt	No. cases	Prevalence (%)
Farrag et al., 1998^13^	1993 to 1994	Assiut Governorate	90/2000	4.5
El Tallawy et al., 2010^14^	2005 to 2009	Al Kharga district, New Valley	180/8173	2.2
El Tallawy et al., 2012^15^	2005 to 2008	Al Kharga district, New Valley	185/8173	2.26
El Tallawy et al., 2013^16^	2009 to 2012	Al‐Quseir city, Red Sea	94/4663	2.01
El Tallawy et al., 2014^17^	2009 to 2012	Al‐Quseir city, Red Sea	87/4329	2.01
Khedr et al., 2015^18^	2011 to 2013	Qena Governorate	35/691	5.07
Rageh et al., 2019^19^	2006 to 2008 + 2009 to 2012	Desert areas, Al Kharga district, and Al Quseir city	126/12,508	1
Awadallah et al., 2020^20^	2017 to 2018	Zagazig city, Sharkia Governorate	108/1820	5.93

Disease registries are proven tools for bridging these gaps, enabling targeted prevention, biomarker discovery, and resource allocation.^11^, ^23^ However, no national dementia registries exist in Africa or the Arab world, and HIC registry models often fail in LMIC settings due to infrastructural and sociocultural barriers.^24^ Recognizing these structural and contextual barriers, we previously proposed a policy framework for LMIC‐friendly dementia registries, prioritizing adaptive diagnostic strategies, population‐based recruitment, and cost‐effective longitudinal data collection.^22^

^22^Here, we implement this framework through the Egyptian Dementia Network (EDN) registry, the first national dementia registry in Africa and the Arab world. The EDN is designed to address three critical gaps in LMIC dementia research and policy: (1) How do sociodemographic factors (e.g., 63% illiteracy among elderly population, multigenerational households) redefine dementia risk and caregiving in LMICs? (2) Can LMIC‐adapted registries uncover actionable biomarkers and comorbidities in understudied populations? And (3) What lessons can be learned for scaling sustainable dementia surveillance and informing policy in resource‐limited settings?

By piloting the EDN registry across six governorates, we aim to establish a multicenter registry model that can be replicable in similar resource‐limited LMICs, challenging HIC‐centric paradigms while advancing equitable dementia care.

**RESEARCH IN CONTEXT**

1. **Systematic review**: We conducted a literature review using Embase, Scopus, PubMed, and relevant regional databases to identify studies on dementia epidemiology, care, and registries in Egypt and comparable LMICs. Most available data are from small, regional studies with heterogeneous methods, and no national dementia registry has been established in Egypt or the wider region.
2. **Interpretation**: Our findings demonstrate that the EDN registry provides the first multicenter, standardized data on dementia in Egypt, revealing late‐stage diagnosis, limited access to care, and unique environmental exposures. This addresses a critical evidence gap and establishes a foundation for national dementia surveillance.
3. **Future directions**: Future research should expand registry coverage for national representativeness, incorporate longitudinal follow‐up, and validate culturally appropriate cognitive assessment tools. Key questions include the impact of environmental exposures on dementia risk and the effectiveness of tailored interventions in resource‐limited settings.

## 2 METHODS

### 2.1 Registry design and implementation

The EDN registry is the first national dementia registry in Egypt, designed as a multicenter, prospective, observational cohort. Phased implementation began in July 2022 at three academic centers (Sohag University, Ain Shams University, and Assiut University), which were the initial collaborators. Subsequently, three additional centers, Mansoura, Aswan, and Beni Suef, were incorporated to ensure broader national representation. The registry's structure and procedures were directly informed by our previously published policy model for LMICs, emphasizing feasibility, scalability, and integration with national dementia policy priorities.^22^

In addition to standardized clinical and sociodemographic data collection, the EDN registry systematically collected biosamples (blood and serum) from participants at each collaborating center. These biosamples were delivered to and stored in the American University in Cairo (AUC) biobank, following standardized protocols for processing and long‐term storage. This biorepository infrastructure enables future genomics analyses and biomarker testing to investigate molecular, genetic, and metabolic contributors to dementia in the Egyptian population, in line with international best practices for population‐based biobanks.^25^ The integration of biosample collection with comprehensive phenotypic data provides a robust platform for multidisciplinary dementia research and supports the development of precision medicine approaches tailored to LMIC settings.

### 2.2 Stakeholder engagement and governance

In line with international best practices and our published policy model, the registry framework was shaped through extensive consultations with national and international experts in neurology, geriatrics, health policy, and registry science. For the practical implementation phase, the development of the data collection sheet involved focused meetings with medical practitioners from the initial collaborating centers to ensure clinical relevance and feasibility. Oversight of registry governance, ethics, and data quality is provided by a steering committee composed of researchers and clinicians from all collaborating centers, ensuring alignment with the World Health Organization Global Dementia Action Plan and local health system needs.

### 2.3 Development of data collection tool

The standardized data collection sheet (Table S1) was collaboratively created with input from medical practitioners at participating centers, as guided by our policy framework. Designed to ensure consistent, high‐quality data capture, the tool covers subjects’ demographics, medical history, diagnostic data, care and treatment, and International Classification of Diseases, 10th Revision, Clinical Modification (ICD‐10‐CM) coding. It was piloted and iteratively refined based on feedback from clinical and research teams to maximize clarity and usability across diverse Egyptian clinical settings.

### 2.4 Participant recruitment and inclusion criteria

Participants were recruited from neurology and geriatric clinics at the participating centers. Inclusion criteria were as follows: (1) age ≥ 18 years; (2) clinical diagnosis of dementia or mild cognitive impairment (MCI) according to The Diagnostic and Statistical Manual of Mental Disorders, 5th edition (DSM‐5) or National Institute on Aging‐Alzheimer's Association criteria, confirmed by a consultant neuropsychiatrist; and (3) provision of informed consent by the participant or a legally authorized representative.

Participation was voluntary, with the right to withdraw at any time. For subjects with advanced cognitive impairment, consent was obtained from a responsible caregiver or legal representative.

### 2.5 Data collection procedures

Data were collected using the standardized EDN data sheet, which includes:

1. Sociodemographic information: age, sex, education, employment, marital status, living arrangements, and urban/rural residence.
2. Medical history: comorbidities, family history of dementia, and lifestyle factors (body mass index [BMI], smoking).
3. Diagnostic data: clinical diagnosis and dementia subtype, classified using ICD‐10‐CM codes (Table S1).
4. Functional assessments:
5. Katz Index of Independence in Activities of Daily Living (ADL): Assesses basic self‐care tasks (bathing, dressing, toileting, transferring, continence, and feeding). Scores range from 0 (dependent) to 6 (independent).
6. Lawton–Brody Instrumental Activities of Daily Living (IADL): Evaluates complex daily tasks (shopping, meal preparation, medication management, finances). Scores range from 0 (dependent) to 8 for women/5 for men (independent).
7. Comprehensive neuropsychological evaluation:
8. Clinical Dementia Rating (CDR) scale: Stages dementia severity (0 = none, 3 = severe) based on memory, orientation, judgment, and function.
9. Psychometric assessment: Includes a brief cognitive test (MMSE or Montreal Cognitive Assessment [MoCA]), with scores adjusted for education level. For low‐literacy participants, informant‐based tools were used where appropriate.
10. Neuroimaging: magnetic resonance imaging or computed tomography scan findings, following standardized protocols (Table S1).
11. Care and treatment: medications, psychosocial support received, and caregiver status.

Data were collected by trained clinicians and research staff at each participating center and then sent to the central team for entry into the registry database. To address the high prevalence of low literacy among participants, cognitive assessments were adapted using validated non‐verbal or informant‐based tools.

### 2.6 Data management and quality assurance

All data are maintained with strict confidentiality and privacy protections. Data were entered into a secure, web‐based platform with regular validation checks to ensure data quality and consistency. Ongoing site monitoring, centralized audits, and harmonization protocols further ensured data integrity and comparability across centers. The registry infrastructure was designed for scalability and future interoperability with electronic health records.

### 2.7 Follow‐up and longitudinal data

Annual follow‐up assessments are planned to monitor disease progression, update clinical and sociodemographic data, and assess outcomes over time.

### 2.8 Data analysis

Descriptive statistics were used to summarize cohort characteristics, including sociodemographic variables, clinical diagnoses, comorbidities, functional and cognitive assessment scores, and environmental exposures. Frequencies and percentages were calculated for categorical variables, while means, standard deviations, and ranges were reported for continuous variables.

### 2.9 Ethical considerations

The study protocol was approved by the institutional review boards of AUC and the participating centers. Written informed consent was obtained from all participants or their legal representatives. The registry adheres to the Declaration of Helsinki and national data protection regulations.

### 2.10 Limitations

Potential limitations include challenges in data collection, diagnostic accuracy for dementia subtypes, and participant recruitment due to stigma or logistical barriers.^26^ As a pilot study, the findings may not be generalizable to the entire Egyptian population but will provide critical initial insights and inform future registry expansion.

## 3 RESULTS

### 3.1 Cohort establishment and data completeness

**FIGURE 1:**
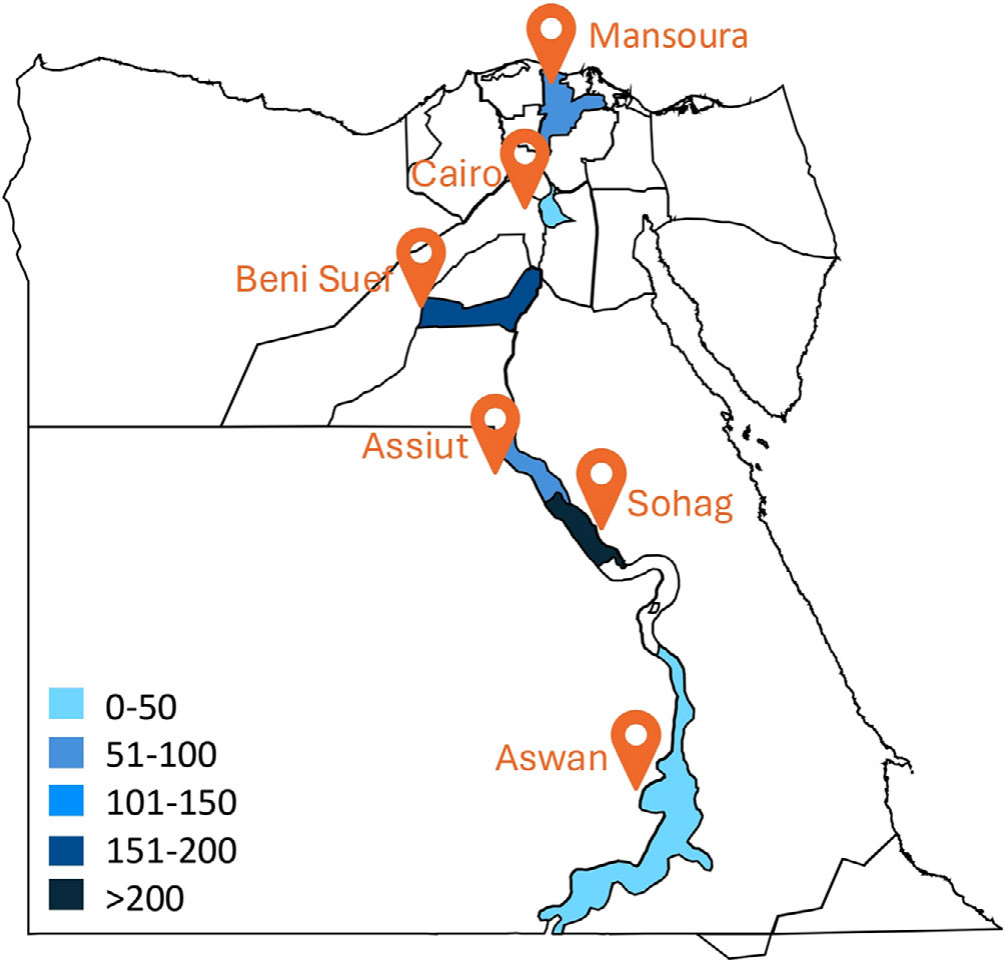
Geographical distribution of collaborating centers and data collected.

A total of 662 participants were recruited across six centers: Sohag (279), Beni Suef (184), Assiut (90), Mansoura (51), Aswan (27), and Ain Shams (31) (Figure 1). Data completeness varied by variable, with sex reported for 661 participants, age for 642, education for 454, and marital status for 346. Most clinical and functional assessments had lower reporting rates, reflecting real‐world data collection challenges in diverse settings.

### 3.2 Demographic characteristics

Table 2 summarizes the demographic characteristics of the EDN registry cohort (*N* = 662), providing critical context for interpreting dementia risk and care needs in Egypt. The sample is nearly evenly divided by sex (50.7% males, 49.3% females), with a mean age of 68.3 years (range 22 to 98), ensuring that the registry captures both early‐ and late‐onset dementia presentations. BMI, reported for 386 participants, averaged 26.2 and ranged from 17.6 to 41.5, reflecting substantial variability in nutritional status and highlighting the coexistence of undernutrition and obesity within the aging population.

**TABLE 2:** Demographic characteristics of Egyptian Dementia Network (EDN) cohort.

Demographic	Reported (*n*)	Group	Total (*n*)	Percentage (%)
Sex	661	Males	335	50.7
		Females	326	49.3
Age	642	Average	68.8	
		Min	22	
		Max	98	
Body mass index	386	Average	26.2	
		Min	17.6	
		Max	41.5	
Education	454	Illiterate	299	65.9
		Grade school	70	15.4
		High school	45	9.9
		College	27	5.9
		Graduate degree	12	2.6
Marital status	346	Single	10	2.9
		Married	250	72.3
		Widow	85	24.6
		Divorced	1	0.3
Employment status	451	Employed	93	20.6
		Unemployed	263	58.3
		Retired	95	21.1
Current address	387	Urban	216	55.8
		Rural	43	11.1
Residence	282	Family home	199	70.6
		Own home	77	27.3
		Nursing home	5	1.8
		Other	1	0.4
Living arrangement	390	With caregiver	376	96.4
		Alone	8	2.1
		Nursing home	5	1.3
		Other	1	0.3
Caregiver	390	Spouse/children	233	59.7
		Other family member	151	38.7
		Formal caregiver	6	1.5

Educational attainment is notably low, with nearly two‐thirds (65.9%) of participants being illiterate and only 2.6% holding a graduate degree. This pronounced skew toward lower education is significant, as it mirrors broader national trends among older Egyptians and underscores the importance of adapting cognitive assessment tools for low‐literacy populations. Marital status data reveal that most participants are currently married (72.3%), while 24.6% are widowed, which refers to both men and women who have lost a spouse. This pattern is consistent with demographic trends in later life, where differences in life expectancy and social support affect both sexes. Employment status shows that the majority are unemployed (58.3%), further illustrating the socioeconomic vulnerabilities common in this age group.

Most participants live in family homes (70.6%), with a smaller proportion residing in their own homes (27.3%) or in care facilities (1.8%). Living arrangement data highlight the centrality of informal caregiving: 96.4% live with a caregiver, most often a spouse or children (59.7%) or another family member (38.7%), while only 2.1% live alone, and formal caregiving remains rare (1.5%). This strong reliance on family networks is a defining feature of dementia care in Egypt.

### 3.3 Lifestyle, social, and functional characteristics

The lifestyle characteristics of the EDN cohort are detailed in Table 3. Among the 542 participants with available data on smoking status, the majority were non‐smokers (84.5%), while 10.7% were current smokers and 4.8% were ex‐smokers. Social activity was reported for 256 participants, with 59% identified as socially active and 41% as not active, indicating that a substantial proportion of the cohort maintains some level of social engagement. Physical activity levels were notably low: of 252 respondents, only 4.4% reported being physically active, while 95.3% reported no regular physical activity. Functional assessments further underscore these lifestyle patterns, with an average IADL score of 3.0 (range: 0 to 8), and an average ADL score of 4.3 (range: 0 to 6) among 195 participants, reflecting considerable limitations in both independent living skills and basic self‐care.

**TABLE 3:** Descriptive statistics of lifestyle characteristics of EDN cohort.

Category	Reported (*n*)	Distribution	Total (*n*)	Percentage (%)
Smoking status	542	Smoker	58	10.7
		Ex‐smoker	26	4.8
		Non‐smoker	458	84.5
Social activity	256	Active	151	59
		Not active	105	41
Physical activity	252	Active	11	4.4
		Not active	241	95.3
IADL score	195	Average	3.01	–
		Minimum	0	–
		Maximum	8	–
ADL score	195	Average	4.31	–
		Minimum	0	–
		Maximum	6	–

Abbreviations: ADL, Activities of Daily Living; IADL, instrumental Activities of Daily Living.

### 3.4 Clinical characteristics and family history

Table 4 presents the clinical characteristics and family history of the EDN cohort. Of the 662 participants with available diagnostic data, the vast majority (92.3%) were diagnosed with dementia, while 7.7% were classified as having MCI. This distribution reflects the registry's focus on capturing the spectrum of cognitive decline, with a predominance of individuals at more advanced stages.

**TABLE 4:** Clinical characteristics and diagnosis of EDN subjects.

Category	Reported (*n*)	Distribution	Total (*n*)	Percentage (%)
Disease	662	Mild cognitive impairment	51	7.7
		Dementia	611	92.3
Family history	382	Positive	85	22.3
		Negative	297	77.4
Age at onset	621	Average	65.4	
		Minimum	14	
		Maximum	94	
Age at diagnosis	582	Average	67.6	
		Minimum	20	
		Maximum	95	
Stage at onset	582	Mild	393	67.5
		Moderate	143	24.6
		Severe	47	8.1
Current stage	581	Mild	280	48.2
		Moderate	214	36.8
		Severe	87	15
Onset type	467	Insidious	270	6.6
		Gradual	166	35.5
		Subacute	31	57.8
Progression speed	376	Slowly progressive	325	86.4
		Rapidly progressive	51	13.6

A family history of dementia was reported for 382 participants. Among these, 22.3% had a positive family history, suggesting a notable genetic or familial component within the cohort, while 77.4% reported no known family history of dementia. This information provides a valuable foundation for future analyses of genetic and environmental risk factors in the Egyptian population.

Age at onset was available for 621 participants, with a mean age of 65.4 years, ranging from as young as 14 to as old as 94. The wide range highlights the inclusion of both early‐ and late‐onset cases within the registry. Similarly, age at diagnosis was reported for 582 participants, with a mean of 67.6 years (range: 20 to 95), indicating a typical diagnostic delay of approximately 2 years from symptom onset to formal diagnosis.

### 3.5 Dementia subtypes and clinical diagnosis

**FIGURE 2:**
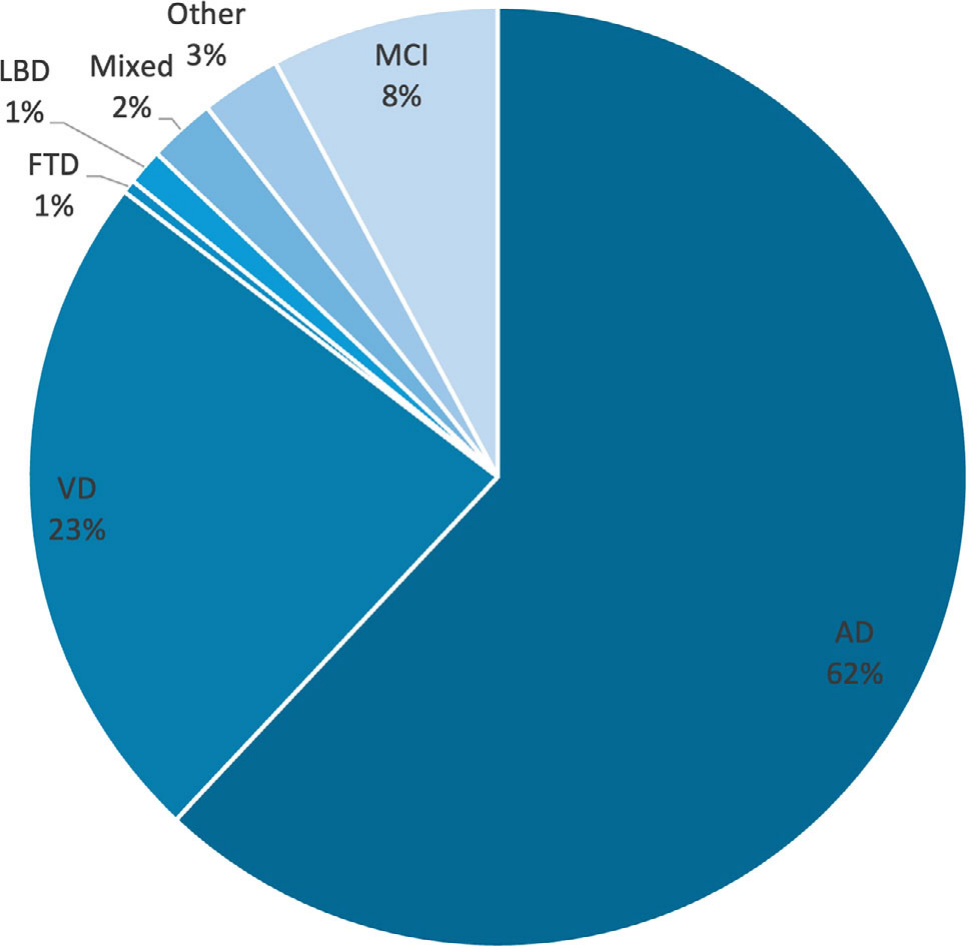
Distribution of dementia subtypes among Egyptian Dementia Network (EDN) cohort.

Further analysis of subjects with dementia (*n* = 611) revealed that AD was the predominant diagnosis, accounting for 62.0% of cases, followed by vascular dementia at 23.4%. Other subtypes – including mixed Alzheimer's and vascular dementia, dementia with Lewy bodies, frontotemporal dementia, and MCI – collectively accounted for a smaller proportion of the cohort. This pattern is consistent with global epidemiological trends, where AD remains the most common form of dementia, but also highlights the significant burden of vascular and mixed dementias in the Egyptian population (Figure 2).

The clinical stage at both symptom onset and current evaluation was also reported. At onset, the majority of participants (67.5%) presented with mild dementia, while 24.6% were classified as moderate and 8.1% as severe. By the time of current assessment, the proportion of participants in the mild stage had decreased to 48.2%, with increases in moderate (36.8%) and severe (15.0%) stages, reflecting disease progression over time. This shift underscores the importance of early detection and intervention, as well as the need for ongoing support as the disease advances (Table 4).

The onset type was predominantly insidious (57.8%), followed by gradual (35.6%) and subacute (6.6%). This distribution aligns with the typical clinical course of neurodegenerative dementias, where symptoms often develop slowly and may go unrecognized in the early stages. Regarding progression speed, most cases were classified as slowly progressive (86.4%), while a minority (13.6%) exhibited a rapidly progressive course. Rapidly progressive dementia, though less common, is clinically significant due to its severe and swift impact on cognitive and functional abilities and may require distinct diagnostic and management strategies (Table 4).

### 3.6 Cognitive assessment

Cognitive screening using the MMSE was completed for 458 participants, with a mean score of 15.6 (range: 0 to 28), indicating moderate to severe cognitive impairment in this population.^27^, ^28^ MoCA scores, available for 83 participants, averaged 19.6 (range: 0 to 27), further supporting the presence of significant cognitive deficits.^29^ The CDR global score, reported for 176 individuals, had a mean of 2.67, consistent with advanced dementia stages, while the CDR sum of boxes (*n* = 54) averaged 9.6, reflecting substantial impairment across multiple domains.^30^ Comprehensive neuropsychological evaluation scores (*n* = 169) were also markedly low (mean: –1.88), underscoring the high burden of cognitive and functional decline in this cohort (Table 5).

**TABLE 5:** Average functional and cognitive scores of EDN subjects.

Measure	Total reported	Average	Minimum	Maximum
Mini‐Mental State Examination score	458	15.62	0	28
Montreal Cognitive Assessment score	83	19.63	0	27
Clinical Dementia Rating (Global CDR)	176	2.67	1	3
CDR (sum of boxes)	54	9.56	3.25	17.5
Comprehensive neuropsychological evaluation	169	−1.88	−2.54	−1

### 3.7 Comorbidities and health status

The EDN cohort exhibits a substantial burden of medical comorbidities. A significant proportion of the cohort experienced multimorbidity, with 136 subjects having more than two comorbid diseases and 47 individuals affected by three or more conditions. This clustering of multiple health issues further underscores the complex medical profiles present within the EDN cohort. Hypertension was the most common condition, reported in 364 participants, followed by diabetes in 155 and cardiovascular or stroke‐related diseases in 135. Hypercholesterolemia was present in 105 individuals, while neurological disorders were reported in 35 and psychotic disorders in 20. Cerebrovascular disease was documented in 23 cases and thyroid disorders in nine. Additionally, 61 participants reported other chronic diseases. Notably, only six out of 209 respondents reported a history of head trauma. This high prevalence of vascular and metabolic comorbidities aligns with global and regional findings linking these conditions to increased dementia risk.

### 3.8 Dementia management and care

Only a small proportion of the EDN cohort received evidence‐based interventions for dementia management. Of the 262 participants with available data, just 24.4% were prescribed medications for dementia, with the vast majority receiving either memantine or donepezil. Both agents are internationally recognized as standard treatments for AD and are frequently used in Egypt for managing moderate to severe dementia symptoms. Despite evidence supporting their efficacy in improving cognition, function, and behavioral symptoms, as well as their inclusion in clinical guidelines, access to these medications remains limited in this population. Even more striking, among 188 participants with reported data, only 2.1% accessed any form of psychosocial intervention.

### 3.9 Environmental exposure

Environmental toxin exposure was systematically assessed in the EDN cohort (Table 5). Of those with available data, 20.4% (134 participants) reported household exposure to chemical agents, making it the most common route of exposure. Insecticide exposure was also frequent, reported by 18.1% (120 participants), while combined exposure to both pesticides and insecticides was noted in 2.4% (16 participants). Direct exposure to pesticides alone was uncommon (0.5%, three participants), and only a single participant reported herbicide exposure. Notably, occupational exposure, primarily among farmers, was reported by just 0.9% (six participants), underscoring that the vast majority of exposures in this cohort occurred in domestic rather than occupational settings. No cases of exposure to fungicides, rodenticides, or fumigants were reported.

## 4 DISCUSSION

The EDN registry represents a foundational advance for dementia research and policy in LMICs, particularly within the Middle East and North Africa (MENA) region. As the first national dementia registry in Africa and the Arab world, EDN addresses a longstanding gap in systematic, population‐level data on dementia, where prior information was largely limited to fragmented or regional studies.^4^ By establishing a multicenter, harmonized infrastructure, the registry enables a more accurate understanding of dementia epidemiology, risk factors, and care needs, providing a model for other LMICs facing similar demographic and healthcare challenges.

A key contribution of the EDN registry is its demonstration of the feasibility and value of integrating dementia surveillance into national health systems in resource‐limited settings. The registry's design, emphasizing stakeholder engagement, standardized data collection, and biosample integration, reflects best practices identified in international registry literature.^31^ This approach not only supports research into the causes and progression of dementia but also informs health service planning and resource allocation, as highlighted in recent reviews.^11^, ^23^ The collaborative governance and commitment to data sharing position Egypt to contribute meaningfully to global dementia research, including genetic, biomarker, and intervention studies.

The public health implications of the EDN registry findings are profound and multifaceted, as the registry was designed for national representativeness, recruiting across diverse governorates. The high illiteracy prevalence reflects population demographics of Egypt, with a 63% illiteracy rate among elderly adults, rather than sampling bias.^32^ The cohort's demographic profile – characterized by advanced age, low educational attainment, and a high proportion of married or widowed individuals – reflects the broader social structure of Egypt and underscores the influence of social determinants such as education, family support, and socioeconomic status on dementia risk and care.^33^, ^34^ The near‐universal reliance on informal family caregiving, coupled with minimal formal support, highlights significant gaps in the social safety net and places a substantial burden on families.^35^, ^36^ The extremely high prevalence of physical inactivity, alongside widespread hypertension and diabetes, points to a population at elevated risk for cognitive decline and underscores the urgent need for public health strategies that address lifestyle factors and promote healthy aging.^37^, ^38^ Chronic comorbidities are highly prevalent, with hypertension, diabetes, and cardiovascular disease affecting a large proportion of participants, making multimorbidity the norm rather than the exception and reinforcing the need for integrated, multidisciplinary approaches to dementia management.^39^, ^40^

Clinically, the registry reveals that most participants present at moderate to severe stages of dementia, with an average diagnostic delay of 2 years, reflecting limited awareness, stigma, and gaps in primary care pathways.^33^, ^34^ These delays result in missed opportunities for early intervention and contribute to the high burden of advanced disease. Furthermore, fewer than one‐quarter of participants receive pharmacological treatment, and only a small minority access interventions, underscoring systemic limitations in healthcare delivery and highlighting the need for culturally tailored interventions, for example, such as cognitive assessment tools adapted for low‐literacy groups and caregiver‐centered support in family‐based households.^41^, ^42^

Environmental risk factors have emerged as a distinctive and urgent concern in the Egyptian context. The registry identified a high prevalence of household insecticide exposure among dementia patients, far exceeding occupational exposures. This is consistent with growing epidemiological evidence linking neurotoxic pesticide exposure to an increased risk of cognitive decline and AD. Recent studies have shown that both occupational and household exposure to pesticides, as well as other environmental toxins, such as air pollution, are associated with a higher risk of dementia and faster cognitive decline.^43^, ^44^ In the MENA region, rapid urbanization and industrialization have increased exposure to air pollution and other environmental hazards, further elevating dementia risk.^45^, ^46^ These findings highlight the importance of integrating environmental health interventions into dementia prevention strategies, a consideration often overlooked in policy and practice.^47^, ^48^ While baseline analysis covered only household exposures, pilot programs now incorporate neuroexposome assessment (heavy metals, satellite‐based pollution data, and pesticide profiling), positioning EDN for comprehensive, longitudinal exposome research.

From a scientific perspective, the EDN registry's infrastructure for biobanking and harmonized data collection enables Egypt to participate in global research efforts, addressing the underrepresentation of LMIC populations in dementia science. The registry's collaborative governance and commitment to data sharing facilitate cross‐national comparisons and meta‐analyses, advancing the understanding of genetic, environmental, and sociocultural determinants of dementia.^49^

### 4.1 Strategic research programs of the registry cohort

EDN not only provides baseline clinical and demographic data but also serves as a platform for a range of strategic, registry‐enabled pilot studies that demonstrate its scalability and scientific potential. These studies encompass multiple omics and environmental dimensions. In genomics research, analysis of apolipoprotein E allele distribution within the cohort revealed a high prevalence of the ε3 allele, observed gender‐associated risk differences, and pronounced geographic variability among participants.^50^ Metabolomics investigations identified significant alterations in lipid and energy metabolism, as well as the presence of pesticide residues in serum, supporting links between environmental exposures and neurodegeneration.^51^ Proteomic studies further characterized inflammation, immunity, and lipid metabolism signatures that distinguish dementia patients in the Egyptian population.^52^ Complementing these molecular analyses, environmental exposome research integrating blood heavy metal quantification and remote sensing‐based air pollution mapping uncovered elevated pollutant burdens among AD patients, with geographic clustering in industrial and high‐traffic regions (unpublished data). Collectively, these multidisciplinary programs validate the registry's role as a comprehensive platform for multi‐omics and exposome‐integrated dementia research in LMIC settings.

### 4.2 Implications for policy and future research

The EDN registry demonstrates the feasibility and value of establishing a national dementia registry in a resource‐limited setting. Our baseline findings reveal advanced disease at presentation, high comorbidity rates, widespread physical inactivity, and significant care gaps, all shaped by social and environmental vulnerabilities, including notable household chemical exposures. These insights underscore the urgent need for earlier detection, integrated care models, workforce training, and targeted public health interventions addressing both traditional and emerging risk factors. Policy priorities emerging from EDN include the integration of illiteracy‐sensitive cognitive screening tools, stronger caregiver support, and the incorporation of environmental health monitoring into dementia prevention programs. An example is our team efforts to validate a culturally adapted version of the Harmonized Cognitive Assessment Protocol (HCAP).^53^

As the registry expands, it will be an increasingly valuable resource for understanding and addressing the unique challenges of dementia care in LMICs while informing both regional and global dementia strategies. Continued investment in surveillance, research, and culturally tailored interventions will be essential for advancing dementia prevention and care in Egypt and similar contexts.

### 4.3 Limitations

Several limitations merit acknowledgment. Data completeness varied across variables as not all variables were available for the entire cohort (e.g., care data were reported for 262/662 participants). This reflects real‐world limitations in documentation and recruitment practices.^54^ However, these missing data may affect generalizability. In addition, environmental exposures beyond pesticide use (e.g., pollution, heavy metals) were not captured in the baseline but are being addressed through ongoing pilot studies. The cross‐sectional nature of the baseline data precludes causal inference, and the current urban overrepresentation may limit generalizability to rural populations. Sustaining participant contact for longitudinal follow‐up will require ongoing effort, particularly given internal migration and socioeconomic instability. Future priorities include expanding recruitment for broader representativeness, implementing longitudinal follow‐up, validating cognitive assessment tools for low‐literacy populations, investigating genetic and biomarker profiles in collaboration with international partners, and pilot testing culturally adapted interventions based on registry findings.

## 5 CONCLUSION

The EDN registry establishes a feasible, nationally representative dementia platform in Egypt, providing baseline characterization while enabling future genetic, molecular, and environmental studies. Pilot projects in genomics, metabolomics, proteomics, and environmental exposomics showcase scalability, positioning EDN to inform dementia prevention, policy development, and equitable care in Egypt and comparable LMICs. By providing critical insights into disease burden, care gaps, and unique risk factors, it supports health system strengthening, prevention strategies, and equitable care delivery. As the registry matures, it will catalyze regional collaboration and innovation, ensuring that the needs and experiences of underrepresented populations inform both national and global dementia agendas.

## CONFLICT OF INTEREST STATEMENT

The authors have nothing to report. Author disclosures are available in the Supporting Information.

## Supporting information



Supporting Information

Supporting Information

## Data Availability

All participants or legal guardians provided written informed consent following the Declaration of Helsinki. All protocols were approved by the Institution Review Board at the American University in Cairo (IRB‐AUC# 2024‐2025‐028). The data presented in this paper reflect the initial cohort and biosample collection from all six centers.
