# Supplementary material for: The Egyptian Dementia Network (EDN): Baseline characteristics from the first dementia registry in an African Arab country
Source: Alzheimers Dement. 2025 Nov 11;21(11):e70770. doi: 10.1002/alz.70770 (PMC12603777; doi:10.1002/alz.70770)
Supplement: Supplementary file 2 — Supporting Information [file ALZ-21-e70770-s002.docx]

**Egyptian Dementia Network (EDN)**

**Patient’s Data Sheet**

**Service Provider**

| Center of Registration (Clinic ID)/ (Center Initials) |  | Date of Registration |  |
| --- | --- | --- | --- |
| **Physician’s Name:** | | | |
| Medical Specialty: **□** Neurology **□** Psychiatry **□** Geriatrics **□** Family/General Practice **□** Internal Medicine **□** Other | | | |

**Patient Information**

| **Patient’s Name** | | **Case ID: (**Center code-Patient sequence code) | | | | |
| --- | --- | --- | --- | --- | --- | --- |
| First Name: | Middle Initial: | Last Name: | | | | |
| Gender: **□** Male **□** Female | National ID Number: | | | | Telephone: | |
| **Place of Birth:** City: ____________________ Country: _______________ **Date of Birth** (Month/Day/Year):  **Education Level: □** Illiterate **□** Grade School **□** High School **□** College **□** Graduate Degree _________________________ | | | | | | |
| **Patient’s Current Address** | | | | | | |
| Street/ Area: | | | City: | Marital Status | |  |
| **Residence**: □ Own home □ Family home □ Nursing home  **With whom does the patient live?**  **□** Alone **□** Private residence with caregiver **□** Nursing home **□** Other  **Is the caregiver:** **□** Spouse **□** Other family member **□** Formal Caregiver **□** Other | | | | | | |
| **Employment Status:** | | **Weight (Kg):** | | **Height (m):** | | |
| **Smoking status:** | | **BMI:** | | | | |
| **Socially Active: □** Yes **□** No  **IADL assessment score:** | | **Physically Active: □** Yes **□** No  **ADL assessment score:** | | | | |
| **Prolonged exposure to contaminants and/or toxins** **□** Pesticides **□** Insecticides **□** Fungicides **□** Herbicides **□** Rodenticides **□** Fumigants **□** Others (Specify ……………)  Where: **□** Home **□** Work **□** Other How long: ……….. | | | | | | |

**Patient’s Medical History**

| **Age at the beginning of Dementia symptoms? (Age at Onset)** | **Age/ timing of medical diagnosis for dementia if done?** |
| --- | --- |
| Family history of neurodegenerative diseases including dementia | Personal medical history or pathological antecedents |
| **Other health conditions: □** Diabetes **□** Hypertension **□** Cholesterol **□** Cardiovascular disease/ Stroke **□** Cerebrovascular disease **□** Neurological disease **□** Psychotic disease **□** Thyroid **□** Other (specify): | |
| **Surgical History:**  **Previous head traumas □** Yes **□** No (Which age:.........) | |

**Diagnostic Data**

| **□Mild Cognitive Impairment (MCI) □ Dementia** | |
| --- | --- |
| **If possible, classify the patient’s dementia (Dementia Subtype) ICD-10 criteria:** | |
| **Clinical Dementia Rating scale:** | |
| **Stage at Diagnosis: □** Early (Mild) **□** Middle (Moderate) **□** Late (Severe) | |
| **Current stage: □** Early (Mild) **□** Middle (Moderate**) □** Late (Severe) | |
| **Type of Onset: □** Insidious  **□** Gradual  **□** Subacute | **Speed of Progression:**  **□** Slowly progressive  **□** Rapidly progressive |
| **Comprehensive neuro-psychological evaluation:** | |
| **Psychometric assessment**  Brief cognitive test (MMSE or MoCA) Score: | |
| **Neuroimaging testing completed**  **□** CT  **□** MRI  **□** MRI dementia protocol (If possible) | |
| **Additional investigations**  **□** ECG  **□** liver function test  **□** kidney function test (blood urea and creatinine)  **□**  CBC  **□** blood sugar curve  **□** thyroid function test (free T3, T4, TSH), lipogram) | **(Optional tests)**  **□** serum uric acid  **□** Vit E  **□** Vit C  **□** Vit B12  **□** Homocysteine  **□** Serum cupper  **□** Serum iron |

**Patient Care and Treatment**

| **Is the patient receiving prescription medication(s) for Dementia? □** No **□** Yes (specify): |
| --- |
| **Other medications (check all that apply)? □** Anti-depressant □ Anti-Psychotic □ Benzodiazepines **□** Other (specify): |
| **Total number of medications?** |
| **Psychosocial interventions (Post-diagnostic Support) □** Yes **□** No **□** Specify:  **□**cognitive stimulation **□**cognitive training **□**activity planning **□**reminiscence **□**aromatherapy **□**music therapy **□**occupational therapy |

**Appendix**

| **LAWTON - BRODY**  **INSTRUMENTAL ACTIVITIES OF DAILY LIVING SCALE (I.A.D.L.)** | | | |
| --- | --- | --- | --- |
| **Scoring:** For each category, circle the item description that most closely resembles the client’s highest functional level (either 0 or 1). | | | |
| **A. Ability to Use Telephone** |  | **E. Laundry** |  |
| 1. Operates telephone on own initiative-looks up and dials numbers, etc. 2. Dials a few well-known numbers 3. Answers telephone but does not dial 4. Does not use telephone at all | 1  1  1  0 | 1. Does personal laundry completely 2. Launders small items-rinses stockings, etc. 3. All laundry must be done by others | 1  1  0 |
| **B. Shopping** |  | **F. Mode of Transportation** |  |
| 1. Takes care of all shopping needs independently 2. Shops independently for small purchases 3. Needs to be accompanied on any shopping trip 4. Completely unable to shop | 1 | 1. Travels independently on public transportation or drives own car 2. Arranges own travel via taxi, but does not otherwise use public transportation 3. Travels on public transportation when accompanied by another 4. Travel limited to taxi or automobile with assistance of another 5. Does not travel at all | 1 |
|  | 0 |  | 1 |
|  | 0 |  |  |
|  |  |  | 1 |
|  | 0 |  |  |
|  |  |  | 0 |
|  |  |  | 0 |
| **C. Food Preparation** |  | **G. Responsibility for Own Medications** |  |
| 1. Plans, prepares and serves adequate meals independently 2. Prepares adequate meals if supplied with ingredients 3. Heats, serves and prepares meals, or prepares meals, or prepares meals but does not maintain adequate diet 4. Needs to have meals prepared and served | 1 | 1. Is responsible for taking medication in correct dosages at correct time 2. Takes responsibility if medication is prepared in advance in separate dosage 3. Is not capable of dispensing own medication | 1 |
|  | 0 |  | 0 |
|  | 0 |  | 0 |
|  | 0 |  |  |
| **D. Housekeeping** |  | **H. Ability to Handle Finances** |  |
| 1. Maintains house alone or with occasional assistance (e.g. "heavy work domestic help") 2. Performs light daily tasks such as dish washing, bed making 3. Performs light daily tasks but cannot maintain acceptable level of cleanliness 4. Needs help with all home maintenance tasks 5. Does not participate in any housekeeping tasks | 1 | 1. Manages financial matters independently (budgets, writes checks, pays rent, bills, goes to bank), collects and keeps track of income 2. Manages day-to-day purchases, but needs help with banking, major purchases, etc. 3. Incapable of handling money | 1 |
|  | 1 |  |  |
|  |  |  | 1 |
|  | 1 |  |  |
|  |  |  | 0 |
|  | 1 |  |  |
|  | 0 |  |  |
| **Score** |  | **Score** |  |
| **Total score**  A summary score ranges from 0 (low function, dependent) to 8 (high function, independent) for women and 0 through 5 for men to avoid potential gender bias. | | |  |

**Appendix**

| **Katz Index of Independence in Activities of Daily Living** | | |
| --- | --- | --- |
| **Activities**  Points (1 or 0) | **Independence**  (1 Point)  **NO** supervision, direction or personal assistance. | **Dependence**  (0 Points)  **WITH** supervision, direction, personal assistance or total care. |
| **BATHING**  Points: | **(1 POINT)** Bathes self completely or needs help in bathing only a single part of the body such as the back, genital area or disabled extremity. | **(0 POINTS)** Need help with bathing more than one part of the body, getting in or out of the tub or shower. Requires total bathing |
| **DRESSING**  Points: | **(1 POINT)** Get clothes from closets and drawers and puts on clothes and outer garments complete with fasteners. May have help tying shoes. | **(0 POINTS)** Needs help with dressing self or needs to be completely dressed. |
| **TOILETING**  Points: | **(1 POINT)** Goes to toilet, gets on and off, arranges clothes, cleans genital area without help. | **(0 POINTS)** Needs help transferring to the toilet, cleaning self or uses bedpan or commode. |
| **TRANSFERRING**  Points: | **(1 POINT)** Moves in and out of bed or chair unassisted. Mechanical transfer aids are acceptable | **(0 POINTS)** Needs help in moving from bed to chair or requires a complete transfer. |
| **CONTINENCE**  Points: | **(1 POINT)** Exercises complete self-control over urination and defecation. | **(0 POINTS)** Is partially or totally incontinent of bowel or bladder |
| **FEEDING**  Points: | **(1 POINT)** Gets food from plate into mouth without help. Preparation of food may be done by another person. | **(0 POINTS)** Needs partial or total help with feeding or requires parenteral feeding. |
| **TOTAL POINTS: SCORING:** 6 = High (*patient independent*) 0 = Low (*patient very dependent* | | |

**Appendix**

**Classification of Disorders by ICD-10-CM codes**

**A81 Creutzfeldt-Jakob disease**

**F01 Vascular dementia**

F01.0 Vascular dementia of acute onset

F01.1 Multi-infarct dementia

F01.2 Subcortical vascular dementia

F01.3 Mixed cortical and subcortical vascular dementia

F01.8 Other vascular dementia

F01.9 Vascular dementia, unspecified

**G10 Huntington’s disease**

**G20 Parkinson’s disease**

**G30 Alzheimer’s disease**

G30.0 Alzheimer’s disease with early onset (onset usually before the age of 65)

G30.1 Alzheimer’s disease with late onset (onset usually after the age of 65)

G30.8 Other Alzheimer’s disease

G30.9 Alzheimer’s disease, unspecified

**G31 Other degenerative diseases of the nervous system, not elsewhere classified**

G31.0 Circumscribed brain atrophy

Pick’s disease

G31.1 Senile degeneration of brain, not elsewhere classified

G31.2 Degeneration of nervous system due to alcohol

Wernicke-Korsakoff syndrome

G31.8 Other specified degenerative disease of nervous system

Grey-matter degeneration

Lewy body disease

G31.9 Degenerative disease of nervous system, unspecified

**Appendix**

**MR imaging protocol and standardized radiological reporting**

Recommended MRI protocols in patients who suffer from dementia include the following MRI sequences:

- *T2-weighted and Fluid-attenuated recovery (Flair) MR sequences*

Optional 2D or 3D; to identify signal abnormalities within the gray and white matter, including hippocampal signal alterations and to determine the degree of vascular damage comprising white matter hyperintensities, lacunes, and post-ischemic parenchymal defects

- *T1-weighted MR sequences*

3D isotropic; to assess the pattern and extent of brain atrophy (including global cortical atrophy (GCA), medial temporal atrophy (MTA) scores and the posterior/parietal atrophy score)

- *Diffusion-weighted MR images* (DWI)

Axial 2D; to display areas with diffusion restriction indicative of acute ischemia, inflammation, or signal alterations with regard to Creutzfeld Jacob disease

- *Susceptibility-weighted MR images* (SWI)

Axial 2D; to represent microbleeds or superficial siderosis.

**Dementia suspected patients, include assessment of the following features:**

| *Global cortical atrophy* | 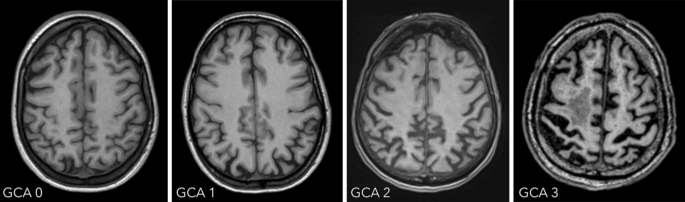 |
| --- | --- |
| *Medial temporal lobe atrophy* | 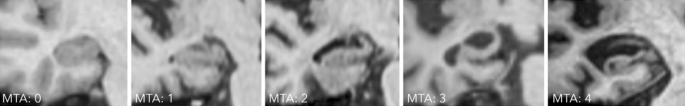 |
| *Posterior/parietal atrophy* | - - 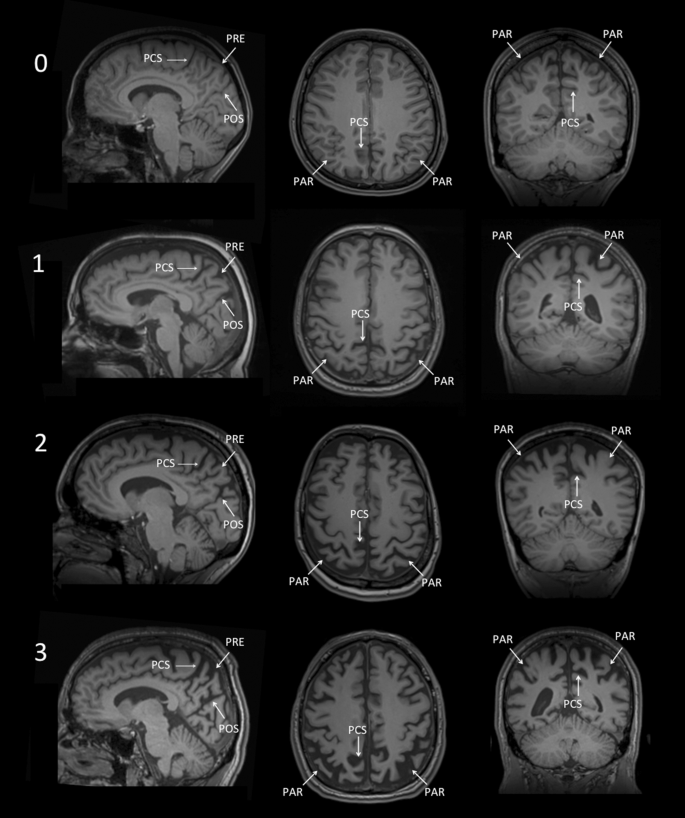 sagittal plane:   precuneus gyrus (PRE)  posterior cingulate sulcus (PCS)  parieto-occipital sulcus (POS)   - - axial plane   posterior cingulate sulcus (PCS)  parietal gyrus (PAR)   - - coronal plane   posterior cingulate sulcus (PCS)  parietal gyrus (PAR) |
| *White matter changes* | 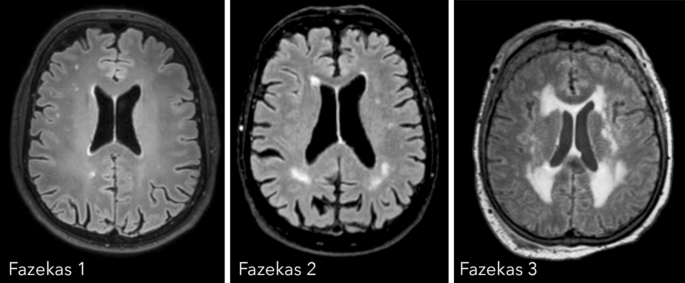 |
| *Lacunes and Virchow-Robin spaces* | 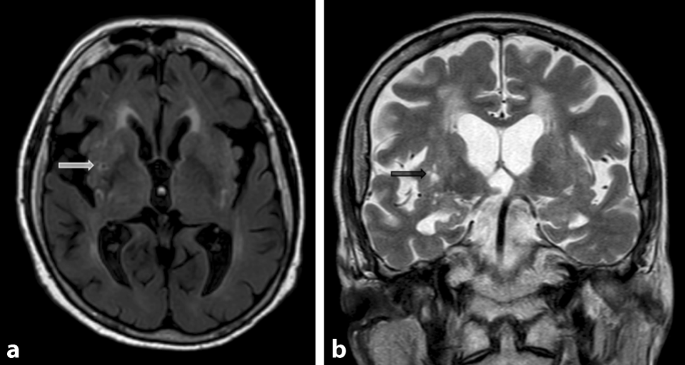 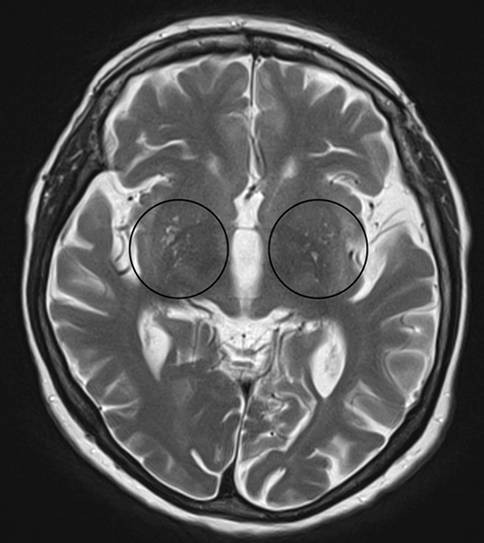 |
| *Microbleeds* | 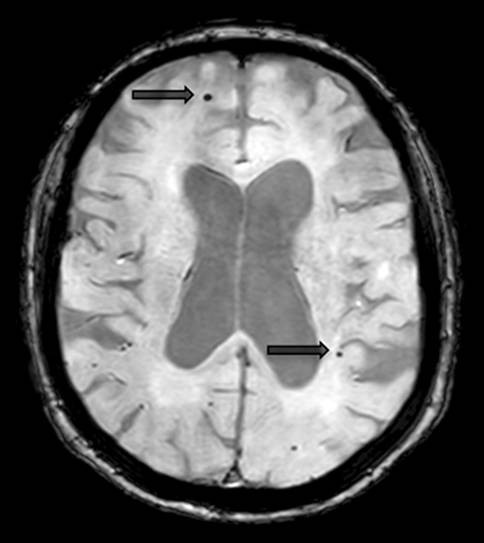 |

## Typical imaging features of the most common dementia subtypes

### Alzheimer’s disease

- **Cortical atrophy** based on underlying neuronal loss, predominantly affects symmetrically the parietal and temporal lobes.
- Late-onset Alzheimer’s disease and patients who present with APOE E4 polymorphism show **hippocampal atrophy**, whereas the central region is relatively spared.
- A significant **asymmetric atrophy** of the hippocampi does not exclude the diagnosis; however, this type of atrophy is more typical for alternative causes, such as frontotemporal dementia.
- Patients with early-onset Alzheimer’s disease or a missing APOE E4 genotype show only **mild or no hippocampal atrophy**, but, instead, present with considerable **posterior cortical atrophy**
- Patients with logopenic progressive aphasia show an **asymmetric more prominent left-sided atrophy** of the posterior temporal cortex and the inferior parietal lobe, which results in language-related impairments.

### Vascular dementia

- Periventricular white matter **hyperintensities**, which can range from punctate to confluent (Fazekas 1–3), cortical/subcortical ischemic/postischemic lesions, lacunar infarcts, enlarged Virchow-Robin spaces, and/or microbleeds.

### Frontotemporal dementia

- Typically **asymmetrical frontal and temporal cortical atrophy**, with a gradient of the imaging findings from anterior to posterior, and a widening of the orbitofrontal sulci as one of the first signs of disease manifestation..
- In patients with progressive non-fluent aphasia, the cortical atrophy is emphasized in the **left-sided anterior** perisylvian region, including the opercular and the insular regions as well as the premotor cortex.
- In patients with semantic dementia, the cortical atrophy is particularly seen in the **anterior and inferior** temporal lobes (ventral and lateral regions).

### Dementia with Lewy bodies

- The medial temporal lobes are relatively preserved in contrast to patients with Alzheimer’s disease.
